# Coordinated Regulation of CB1 Cannabinoid Receptors and Anandamide Metabolism Stabilizes Network Activity during Homeostatic Downscaling

**DOI:** 10.1523/ENEURO.0276-22.2022

**Published:** 2022-11-10

**Authors:** Michael Ye, Sarah K. Monroe, Sean M. Gay, Michael L. Armstrong, Diane E. Youngstrom, Fabio L. Urbina, Stephanie L. Gupton, Nichole Reisdorph, Graham H. Diering

**Affiliations:** 1 Department of Cell Biology and Physiology, University of North Carolina at Chapel Hill, Chapel Hill, NC 27599; 2 University of North Carolina Neuroscience Center, Chapel Hill, NC 27599; 3 Skaggs School of Pharmacy and Pharmaceutical Sciences, University of Colorado Anschutz Medical Campus, Aurora, CO 80045; 4 Carolina Institute for Developmental Disabilities, Carrboro, NC 27510

**Keywords:** anandamide, cannabinoid receptor, endocannabinoid system, homeostatic scaling, network activity, synaptic plasticity

## Abstract

Neurons express overlapping homeostatic mechanisms to regulate synaptic function and network properties in response to perturbations of neuronal activity. Endocannabinoids (eCBs) are bioactive lipids synthesized in the postsynaptic compartments to regulate synaptic transmission, plasticity, and neuronal excitability primarily through retrograde activation of presynaptic cannabinoid receptor type 1 (CB1). The eCB system is well situated to regulate neuronal network properties and coordinate presynaptic and postsynaptic activity. However, the role of the eCB system in homeostatic adaptations to neuronal hyperactivity is unknown. To address this issue, we used Western blotting and targeted lipidomics to measure adaptations in eCB system to bicuculline (BCC)-induced chronic hyperexcitation in mature cultured rat cortical neurons, and used multielectrode array (MEA) recording and live-cell imaging of glutamate dynamics to test the effects of pharmacological manipulations of eCB on network activities. We show that BCC-induced chronic hyperexcitation triggers homeostatic downscaling and a coordinated adaptation to enhance tonic eCB signaling. Hyperexcitation triggers first the downregulation of fatty acid amide hydrolase (FAAH), the lipase that degrades the eCB anandamide, then an accumulation of anandamide and related metabolites, and finally a delayed upregulation of surface and total CB1. Additionally, we show that BCC-induced downregulation of surface AMPA-type glutamate receptors (AMPARs) and upregulation of CB1 occur through independent mechanisms. Finally, we show that endocannabinoids support baseline network activities before and after downscaling and is engaged to suppress network activity during adaptation to hyperexcitation. We discuss the implications of our findings in the context of downscaling and homeostatic regulation of *in vitro* oscillatory network activities.

## Significance Statement

Neurons are remarkably resilient to perturbations in network activities thanks to the expression of overlapping homeostatic adaptations. In response to network hyperactivity or silencing, neurons respond through regulating excitatory and inhibitory postsynaptic neurotransmitter receptors density, probability of presynaptic neurotransmitter release, and/or membrane excitability. The endocannabinoid (eCB) system is a prominent signaling pathway at many synapses that is known to be involved in multiple forms of short-term and long-term synaptic plasticity. Here, we find that components of the endocannabinoid system are upregulated in response to chronic hyperexcitation of cultured cortical neurons, and that endocannabinoid signaling is required to maintain network activity but also suppresses network events during hyperexcitation. This work supports a novel tonic homeostatic function for the endocannabinoid system in neurons.

## Introduction

Neurons express overlapping homeostatic mechanisms to maintain excitability and network properties in response to changes in synaptic inputs. Homeostatic scaling is a well-described bidirectional phenomenon that broadly controls the strength of excitatory and inhibitory synapses through regulation of presynaptic and postsynaptic functions (Turrigiano et al., 1998; Turrigiano, 2008). In response to prolonged hyperexcitation or silencing, homeostatic mechanisms are engaged in cortical and hippocampal neurons to restore activity levels to a set point through compensatory regulation of postsynaptic AMPARs density (O’Brien et al., 1998; Diering et al., 2014), GABAergic transmission (Turrigiano, 2008), and/or presynaptic vesicle release (De Gois et al., 2005; Wierenga et al., 2006). The expression locus of homeostatic mechanisms varies depending on the neuronal types and maturation, with additional presynaptic mechanisms emerging approximately three weeks *in vitro* in cultured primary cortical/hippocampal neurons (De Gois et al., 2005; Wierenga et al., 2006; Turrigiano, 2008). Whereas many molecular mechanisms have been described underlying presynaptic and postsynaptic homeostatic adaptations, it is not entirely clear how presynaptic and postsynaptic glutamatergic and GABAergic plasticity mechanisms are coordinated to maintain stable network activity at baseline and in response to network hyperexcitation.

Endocannabinoids (eCBs) are bioactive lipids that regulate neurotransmitter release and synaptic plasticity, largely through activation of presynaptic cannabinoid receptor type 1 (CB1; Castillo et al., 2012). CB1 is broadly expressed in many neuron types and is known to regulate glutamatergic and GABAergic transmission, primarily through a Gα_i_-coupled suppression of synaptic vesicle release (Castillo et al., 2012; Di Marzo, 2018). CB1 is also known to regulate cortical up-states, a prominent mode of slow oscillatory network activity seen in isolated cortical slices or dissociated cultures *in vitro*, or during nonrapid eye movement (NREM) sleep *in vivo* (Steriade et al., 1993a; Johnson and Buonomano, 2007; Pava et al., 2014; Saberi-Moghadam et al., 2018). Two major eCBs, 2-arachidonyl glycerol (2-AG), and arachidonoyl ethanolamide (AEA; also called anandamide) may act as agonists for CB1, but they are synthesized and degraded through completely nonoverlapping sets of enzymes, suggesting that they may serve distinct functions and undergo separate regulation (Di Marzo, 2018). Unlike other neuromodulators, eCBs are not stored in vesicles for release, but are synthesized “on demand” from the catabolism of postsynaptic phospholipids in an activity-dependent manner. The eCB system constitutes an important retrograde signaling mechanism whereby activity of postsynaptic neurons can regulate presynaptic transmitter release (Castillo et al., 2012). Therefore, the eCB system is ideally situated to coordinate the activities of presynaptic and postsynaptic plasticity and regulate network activity during homeostatic scaling.

In the current study, we have observed that in relatively mature cortical cultures, CB1 protein expression and cell-surface targeting are upregulated in response to chronic (48 h) hyperexcitation induced by the GABA_A_ antagonist bicuculline (BCC). This adaptation is coordinated with a downregulation of fatty acid amide hydrolase (FAAH), and consequent accumulation of AEA and related N-acylethanolamides (NAEs) to enhance tonic CB1-AEA signaling. We show that the well-described downregulation of surface AMPARs during downscaling is independent of CB1 signaling. Using multielectrode array (MEA) recordings and live-cell imaging of a fluorescent glutamate neurotransmitter reporter (iGluSnFr; Marvin et al., 2018), we observe highly synchronized up-state-like oscillatory network activities in dissociated neuron culture (Steriade et al., 1993a; Pava et al., 2014; Saberi-Moghadam et al., 2018). Consistent with a previous report (Pava et al., 2014), we show that CB1 signaling is required to maintain this up-state-like network activity *in vitro*, both under control conditions and following BCC-induced downscaling. Additionally, we show that enhancing CB1 further suppresses *in vitro* network activities during hyperexcitation. We propose that coordinated regulation of eCB metabolism and signaling is needed to adapt and maintain *in vitro* network activity during the response to network hyperexcitation.

## Materials and Methods

### Primary neuron culture

Primary dissociated cortical neurons were prepared from E18 Sprague Dawley rats of both male and female sexes (https://www.criver.com/products-services/find-model/cd-sd-igs-rat?region=3611) as previously described (Diering et al., 2014). Briefly, dissociated cortical neurons were plated onto tissue culture dishes coated with 1 mg/ml poly-L-lysine (PLL; Sigma-Aldrich). Neurons were maintained in at 37°C/5% CO_2_, and fed twice a week in glial conditioned neurobasal media (Invitrogen) supplemented with 2% B27, 1% horse serum, 2 mm GlutaMAX, and 100 U/ml penicillin/streptomycin (Invitrogen). For biochemistry experiments, neurons were plated at 600,000 cells/well into six-well tissue culture plates (Falcon). For lipidomics experiments, neurons were plated at 5,000,000 cells/plate into 10-cm tissue culture dishes (Corning). For live-cell imaging, neurons were plated at 120,000 cells/well into 4-well chambered cover glass (Cellvis). For MEA recording, neurons were plated at 120,000 cells/wells into 24-well Cytoview MEA plates (Axion Biosystems).

### Drug treatment

**Figure 1.**
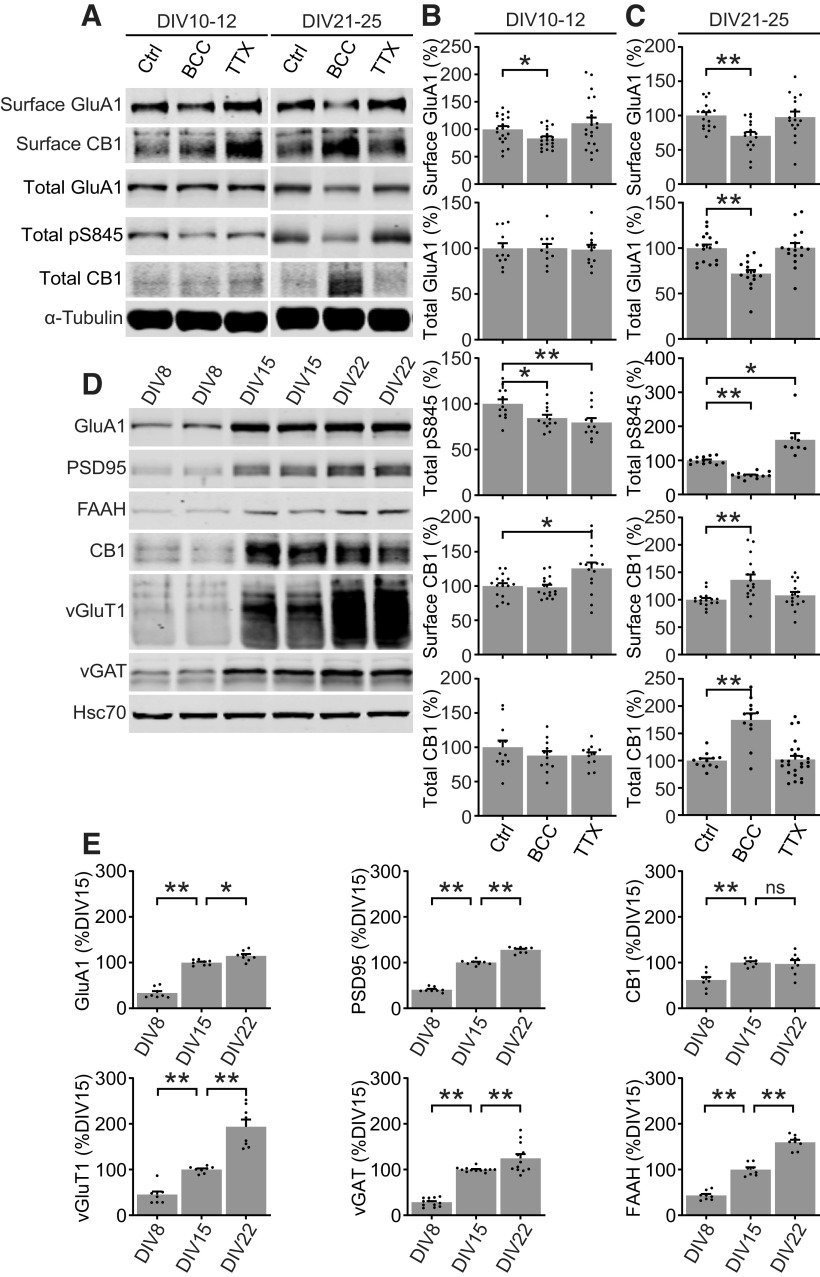
CB1 expression is upregulated during homeostatic downscaling in mature neurons. ***A***, Representative Western blottings of surface and total protein expression in developing (DIV10–DIV12) and mature (DIV21–DIV25) cultured cortical neurons treated for 48 h with control media (Ctrl), 20 μm bicuculline (BCC), or 1 μm tetrodotoxin (TTX). ***B***, ***C***, Quantification of data shown in ***A*** in developing neurons (***B***) and mature neurons (***C***). BCC induced downregulation of surface GluA1 and dephosphorylation of S845 regardless of age, but upregulation of total and surface CB1 only in mature neurons. Results were normalized for each protein to its expression under control levels and presented as mean ± SEM from at least three independent culture preparations with triplicate wells. ***D***, ***E***, Representative Western blottings and quantification of total protein expression in cultured cortical neurons at DIV8, DIV15, and DIV22. Results are normalized for each protein to its expression levels at DIV15, and presented as mean ± SEM from at least three independent culture preparations with quadruplicate wells. Standard unpaired *t* test was used for ***B***, ***C*** and one-way ANOVA with Dunnett’s multiple comparison test was used for ***E***; *p ≤ 0.05, **p ≤ 0.01. ns = not significant.

**Figure 2.**
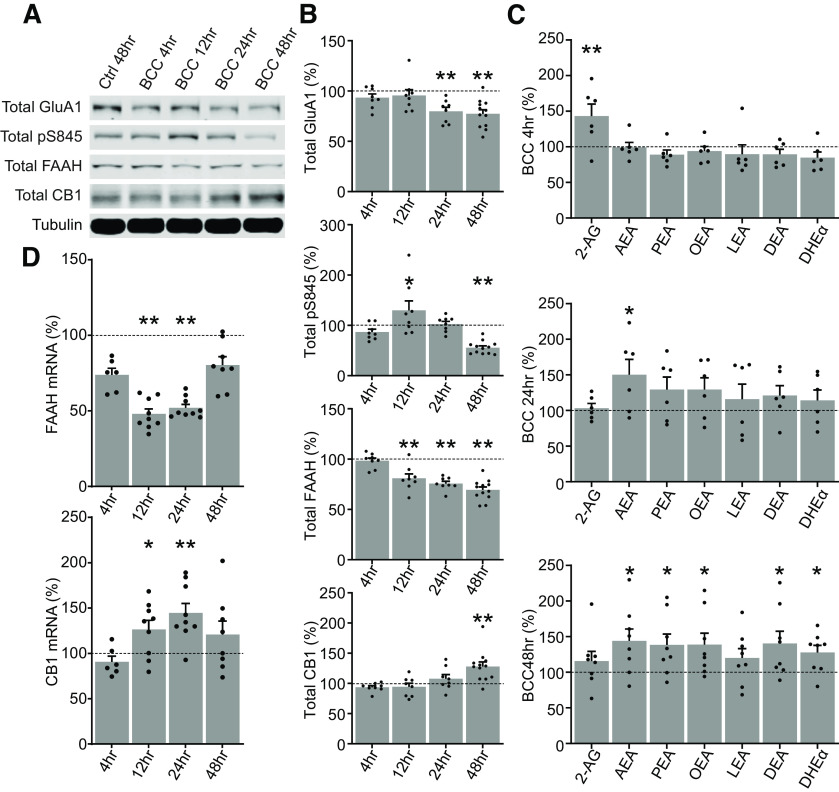
Time course of homeostatic downscaling in rat primary cortical neuron culture. ***A***, ***B***, Representative Western blottings and quantification of total protein expression in DIV17 neurons treated by control media for 48 h (Ctrl 48 h) or by 20 μm bicuculline (BCC) for 4, 12, 24, or 48 h. FAAH is significantly downregulated from 12 h and onward, but CB1 do not become significantly upregulated until the end of the 48-h time course. Results are normalized for each protein to its expression levels in vehicle 48 h (dotted line), and presented as mean ± SEM from four to six independent culture preparations with duplicate wells. ***C***, Quantification of targeted lipidomics analyzing 2-AG, AEA, and various n-acylethanolamines (NAEs) in DIV17 neurons untreated or treated by 20 μm BCC for 4, 24, or 48 h. Note that the neurons transition from a 2-AG dominated response at 4 h to an AEA-dominated and NAEs-dominated response at 24 and 48 h, corresponding with the downregulation of FAAH shown in ***B***. Results from each treatment group are normalized to a matching control and presented as mean ± SEM from three to four independent culture preparations with duplicate plates. ***D***, Quantification of mRNA expression of FAAH and CB1 in DIV18–DIV20 neurons untreated or treated by 20 μm BCC for 4, 24, or 48 h. Results are normalized for each mRNA target to its expression levels in vehicle 48 h (dotted line), and presented as mean ± SEM from three independent culture preparations with triplicate wells. One-way ANOVA with Dunnett’s multiple comparison test; *p ≤ 0.05, **p ≤ 0.01.

**Figure 3.**
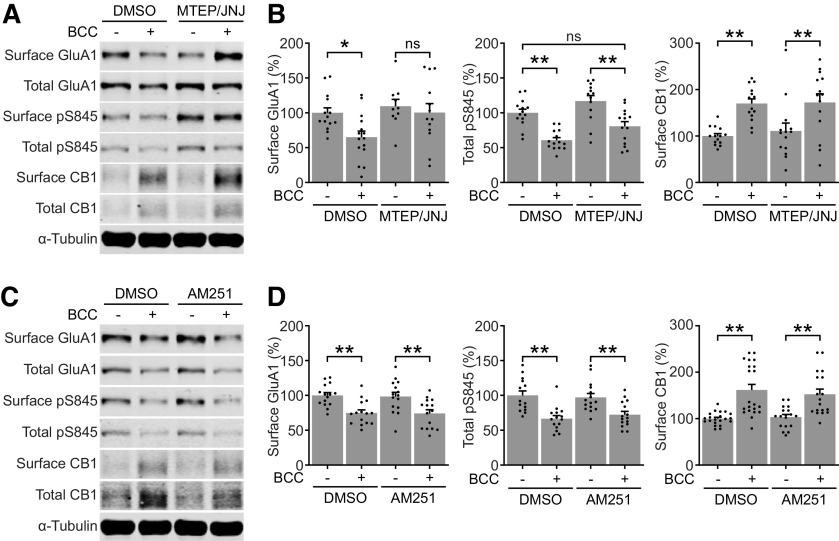
Glutamatergic and cannabinergic adaptations occur through independent mechanisms. ***A***, ***B***, Representative blots and quantifications of surface and total protein expression in DIV21–DIV25 neurons treated by DMSO or 20 μm BCC in the absence or presence of 1 μm MTEP hydrochloride, a selective mGluR5 antagonist, and 100 nm JNJ 16259685, a selective mGluR1 antagonist (MTEP/JNJ). MTEP/JNJ blocked BCC-induced downregulation of surface GluA1 but not the upregulation of surface CB1. ***C***, ***D***, Representative blots and quantifications of surface and total protein expression in DIV21–DIV25 neurons treated by DMSO or 20 μm bicuculline (BCC) in the absence or presence of 500 nm AM251, a selective CB1 antagonist. AM251 did not block BCC-induced downregulation of surface GluA1 or the upregulation of surface CB1. Results are normalized for each protein to its expression levels in control group, and presented as mean ± SEM from four to six independent culture preparations with triplicate wells. One-way ANOVA with Šidák’s multiple comparison test; *p ≤ 0.05, **p ≤ 0.01. ns = not significant.

**Figure 4.**
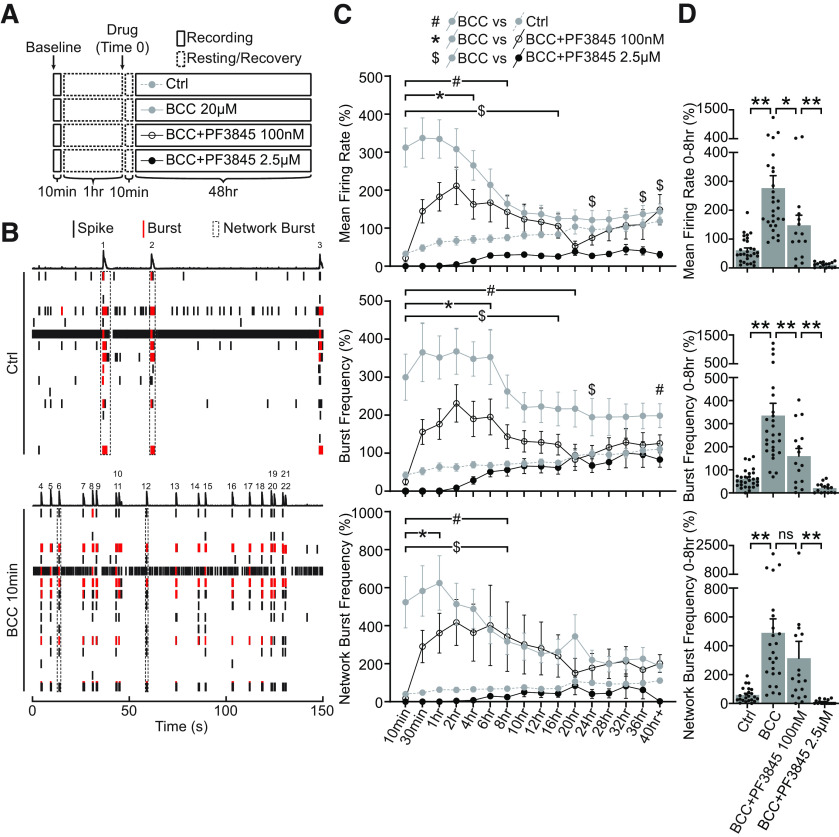
Elevated FAAH-substrates suppress network activities during homeostatic adaptation to BCC-induced hyperexcitation. ***A***, Experimental design. Neurons DIV21–DIV28 were recorded for a 10-min baseline, and then allowed to rest for 1 h. Network activities were then recorded for 10 min once every 30 min over 48 h in neurons treated with ctrl media (Ctrl), 20 μm BCC alone (BCC 20 μm), or 20 μm BCC in combination with PF3845, a potent FAAH inhibitor, at a lower dosage (BCC+PF3845 100 nm) or a higher dosage (BCC+PF3845 2.5 μm). Media were allowed to equilibrate for 10 min after drug treatment (Time 0) before the first point was taken. ***B***, Raster plot of one well (16 electrodes) of MEA activities recorded for 150 s at baseline (Ctrl) or 10 min following 20 μm BCC treatment (BCC 10 min). Single electrode spikes (short black line) and single electrode bursts (short red line) are quantified as mean firing rate and burst frequency, while network bursts (dotted black box or numbered peaks) are quantified as network burst frequency, as shown in ***C***. Note that 20 μm BCC rapidly induces hyperexcitation in network activities within 10 min. This is exemplified here by three network bursts under control, but 19 after 10 min of BCC. ***C***, Quantification of mean firing rate, burst frequency and network burst frequency, as illustrated in ***B***, for neurons DIV21–DIV28 treated for 48 h with ctrl media, 20 μm BCC, BCC+PF3845 100 nm or BCC+PF3845 2.5 μm, as illustrated in ***A***. BCC treatment rapidly (<10 min) converts the network to a burst firing pattern which remains significantly elevated for ∼8 h, and the hyperexcitation can be suppressed by PF3845 in a dose-dependent manner. Results were normalized to a within-well control recorded for 10 min at ∼1 h before treatment (***A***, baseline), and presented as mean ± SEM from six independent culture preparations with quadruplicate wells. Two-way ANOVA with Dunnett’s multiple comparison test #*p* ≤ 0.05 Ctrl versus BCC, *p ≤ 0.05 BCC versus BCC+PF3845 100 nm, $*p* ≤ 0.05 BCC versus BCC+PF3845 2.5 μm throughout the duration indicated by the bracket and/or at time points denoted by the symbols. ***D***, Quantification of average mean firing rate, burst frequency, and network burst frequency for the first 8 h of treatment. Note that the dose-dependent suppression of network activities is particularly prominent in the first 8 h during which the BCC-induced hyperexcitation remains statistically significant. One-way ANOVA with Tukey’s multiple comparison test; *p ≤ 0.05, **p ≤ 0.01. ns = not significant.

**Figure 5.**
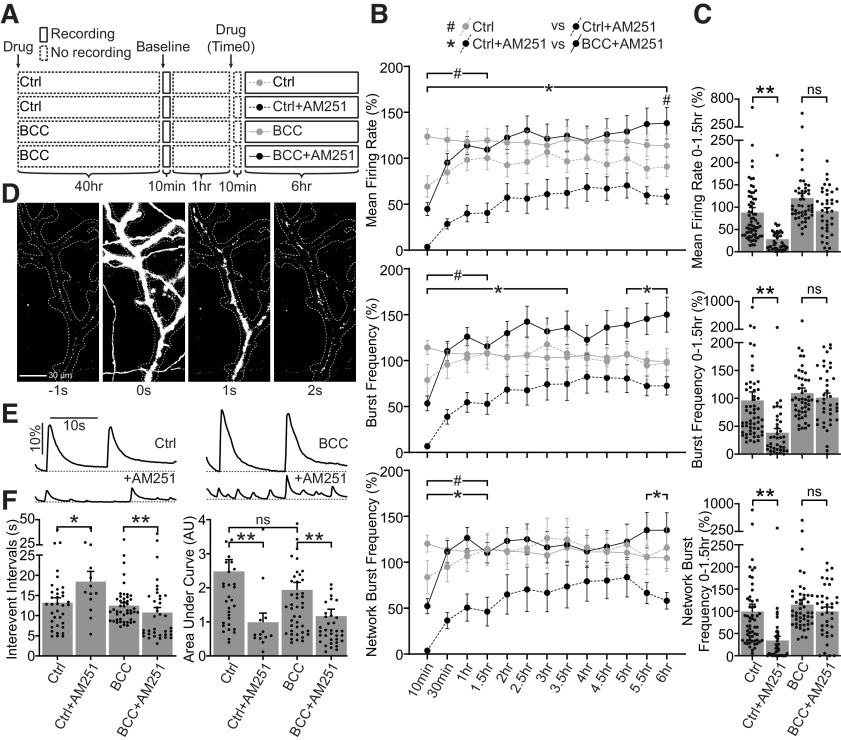
CB1 antagonism suppresses network activities before and after adaptation to BCC-induced hyperexcitation. ***A***, Experimental design. Neurons DIV21–DIV28 were treated with ctrl media (Ctrl, or naive) or 20 μm BCC (BCC, or downscaled) for 40 h before baseline was recorded for 10 min. After resting for 1 h, naive or downscaled neurons were recorded and quantified with MEA in 10-min bins once every 30 min over 6 h in the absence (Ctrl, BCC) or presence (Ctrl+AM251, BCC+AM251) of 50 nm AM251, a potent CB1 inverse agonist. Media were allowed to equilibrate for 10 min after the drug treatment (Time 0) before the first time point was taken. The same experimental design was used for live-cell imaging, but those cells were only recorded for 1 h in the absence or presence of 500 nm AM251. ***B***, Quantification of mean firing rate, burst frequency and network burst frequency from naive neurons untreated (Ctrl) or treated for 6 h with 50 nm AM251 (Ctrl+AM251), and from downscaled neurons untreated (BCC) or treated for 6 h with 50 nm AM251 (BCC+AM251), as illustrated in ***A***. Note that AM251 strongly suppresses network activities in naive cells which remains significantly depressed for 1.5 h, but the response is greatly attenuated in downscaled cells. Results were normalized to a within-well control recorded for 10 min at ∼1 h before treatment (***A***, baseline**)**, and presented as mean ± SEM from four to six independent culture preparations with quadruplicate wells. Two-way ANOVA with Tukey’s multiple comparison test; #*p* ≤ 0.05 Ctrl versus Ctrl+AM251, *p ≤ 0.05 Ctrl+AM251 versus BCC+AM251 throughout the duration indicated by the bracket and/or at time points denoted by the symbols. ***C***, Quantification of average mean firing rate, burst frequency and network burst frequency for the first 1.5 h of treatment, during which AM251-induced suppression of network activities remains statistically significant in naive cells. Note that attenuation of AM251-induced suppression of network activities is particularly prominent during the first 1.5 h. ***D***, Double derivative images showing spontaneous changes in iGluSnFR fluorescence corresponding to a single synchronous glutamate event under control condition. ***E***, Representative traces of iGluSnFR fluorescence corresponding to multiple synchronous glutamate events recorded in DIV21–DIV25 culture cortical neurons naive and untreated (Ctrl, top left), naive and treated with 500 nm AM251 for up to 1 h (Ctrl+AM251, bottom left), downscaled and untreated (BCC, top right) or downscaled and treated with 500 nm AM251 for up to 1 h (BCC+AM251, bottom right), similar to MEA experimental design illustrated in ***A***. Results are presented as change in fluorescence for each recording normalized to its minimum fluorescence (ΔF/F, dotted line represent minimum fluorescence). Scale bar represents 10 s (horizontal) and 10% change from minimum fluorescence (vertical). Ctrl data in panel are from the same set of data as in Extended Data Figure 5-1. ***F***, Quantification of interevent intervals and area under curve for data shown in ***E***. Acute AM251 application causes reduction of glutamatergic event amplitude in naive and downscaled cells but leads to increased frequency in downscaled and reduced frequency in naive cells. Results are presented as mean ± SEM from three to six independent culture preparations with four to eight wells in each culture. One-way ANOVA with Šidák’s multiple comparison test; *p ≤ 0.05, **p ≤ 0.01. ns = not significant.

Drugs were prepared as 1000× stocks and stored at −20°C. To induce homeostatic scaling, neurons were treated with 20 μm bicuculline methobromide (BCC; Tocris, 20 mm stock in water) or 1 μm tetrodotoxin citrate (TTX; Abcam, 1 mm stock in water) for 48 h (Fig. 1). In some live-cell imaging experiments, 20 μm BCC or 1 μm TTX was applied acutely for 10–60 min. For Figure 2, neurons were treated with 20 μm BCC for 4, 12, 24, or 48 h to monitor the process of homeostatic down-scaling. For Figure 3, neurons were treated for 48 h with a combination of 1 μm MTEP hydrochloride (Tocris, 1 mm stock in DMSO) and 100 nm JNJ 16259685 (Tocris, 100 μm stock in DMSO) to block mGluR1/5 signaling. For Figure 4, neurons were treated for 48 h with 100 nm PF3845 (Cayman, 100 μm stock in DMSO) or 2.5 μm PF3845 (Cayman, 2.5 mm stock in DMSO) to inhibit FAAH and to elevate AEA and related NAEs. For Figure 5, neurons were treated for 6 h with 50 nm AM251 (Cayman, 50 μm stock in DMSO), or for 10–60 min with 500 nm AM251 (Cayman, 500 μm stock in DMSO) to block CB1 signaling. For Extended Data Figure 5-1, neurons were treated for 10–60 min with 50 μm D-AP5 (Cayman, 50 mm stock in water) to block NMDA signaling.

10.1523/ENEURO.0276-22.2022.f5-1 Extended Data Figure 5-1 Fluorescence responses of iGluSnFR synchronous events to acute (10 min) application of pharmacological treatments: Ctrl (***A***), 20 μm BCC (***B***), 50 μm D-AP5 (***C***), and 1 μm TTX (***D***). Note the similarity to previously reported MEA network activities: glutamatergic events show hyperexcitation to BCC, a GABA antagonist, is dramatically reduced but not eliminated by D-AP5, an NMDA antagonist, and completely abolished by TTX, a voltage-gated sodium channel blocker. Results are presented as change in fluorescence for each recording normalized to its minimum fluorescence (ΔF/F, dotted line represent minimum fluorescence). Scale bar represents 10 s (horizontal) and 10% change from minimum fluorescence (vertical). Ctrl data in panel are from the same set of data as in Figure 5. Download Figure 5-1, EPS file.

### Antibodies

The following mouse primary antibodies were used: anti-GluA1 (NeuroMab 75-327, 1:1000), anti-FAAH (Abcam 1:1000), anti-PSD95 (NeuroMab 75–028, 1:1,000,000) and anti-α-Tubulin (Abcam, 1:50,000). The following rabbit primary antibodies were used: anti-GluA1 phospho-S845-specific (Millipore 1:1000) and anti-CB1 (Cell Signaling, 1:1000). The following guinea pig primary antibodies were used: anti-vGluT1 (Synaptic Systems 135304, 1:11,000), anti-vGAT (Synaptic Systems 131005, 1:1000). The following secondary antibodies were used: goat anti-mouse 680RD (Licor, 1:10,000), goat anti-rabbit 800CW (Licor, 1:15,000), and donkey anti-guinea pig 800CW (Licor, 1:15,000).

### Cell surface biotinylation

Surface biotinylation of cultured cortical neurons were performed as previously described (Diering et al., 2014). Briefly, neurons were rinsed with ice-cold PBSCM (PBS containing 0.1 mm CaCl_2_ and 1 mm MgCl_2_, pH 8.0), incubated in PBSCM containing 0.5 mg/ml Sulfo-NHS-SS-biotin (Thermo Scientific, 30 min, 4°C), then rinsed in ice-cold PBSCM. Excess biotin was then quenched twice in PBSCM containing 20 mm glycine (2 × 7 min, 4°C), and then the cells were washed again with ice-cold PBSCM. Cells were lysed in ice-cold lysis buffer (PBS containing 1% Triton X-100, 0.5% sodium deoxycholate, 0.02% SDS, 1 mm EDTA, 5 mm sodium pyrophosphate, 1 μm okadaic acid, 1 mm Na_3_VO_4_ and phosphatase inhibitor cocktail; Roche). Lysates were precleared with centrifugation (17,000 × *g*, 10 min, 4°C). Protein concentration of each precleared lysate was quantified using Bradford reagent (Bio-Rad), and equal amounts of proteins were incubated overnight with NeutrAvidin-coupled agarose beads (Thermo Scientific). Beads were washed three times with ice-cold PBS containing 1% Triton X-100, and biotinylated proteins were eluted with 2× SDS sample buffer at 65°C for 20 min. Cell surface or total proteins were then subjected to SDS-PAGE and analyzed by Western blotting.

### Western blotting

Lysates from cultured cortical neurons were precleared with centrifugation (17,000 × *g*, 10 min, 4°C). Protein concentration was determined using Bradford assay. Precleared lysates were mixed with 2× SDS sample buffer (20% glycerol, 100 mm Tris, 5% SDS, 5% BME, pH 6.8) and denatured at 65°C for 20 min. Equal amounts of proteins were loaded and separated by SDS-PAGE on hand-cast gels. Separated proteins were transferred to a nitro-cellulose membrane (GE Healthcare). Following transfer, membranes were blocked in 3% BSA in TBS for 45 min at room temperature. After blocking, membranes were incubated with primary antibodies dissolved in TBST (TBS containing 0.1% Tween 20) containing 3% BSA, overnight at 4°C. Membranes were then washed in TBST (3× 15 min) before being incubated in secondary antibodies (Licor) dissolved in TBST containing 3% BSA and 0.01% SDS for 1 h at room temperature. Membranes were then washed again in TBST (3× 40 min). Membranes were imaged on Licor Odyssey CLx Imaging System. Blots were analyzed and quantified using Image Studio software (Licor).

### Targeted mass spectrometry

Rat primary cortical neurons were grown on 10-cm tissue culture plates at a density of 5,000,000 cells/plate for 17 days *in vitro* (DIV). Culture media were aspirated, cells were rinsed with ice cold PBS and then collected and suspended by scraping in PBS containing 50 nm JZL195 (Tocris), a dual inhibitor of FAAH and MAGL (Long et al., 2009). Cells were pelleted by centrifugation at 5000 × *g* for 5 min, the supernatant was removed and cell pellets were flash frozen and stored at −80°C until further analysis. Frozen cell pellets were prepared for endocannabinoid analysis as follows. Briefly, cell pellets were removed from −80°C freezer and thawed on ice. To each sample 170 μl of methanol, 20 μl of internal standard containing 200 ng/ml each of arachidonyl ethanolamide-d4 and Oleoyl ethanolamide-d4 and 2000 ng/ml of 2-arachidonlyl glycerol-d5, and 10 μl of 5 mg/ml BHT in ethanol was added. The cell pellet was resuspended and then vortexed for 5 s. The sample was then centrifuged at 14,000 RPM for 10 min at 4°C. The supernatant was removed and then placed into a capped autosampler vial for analysis. Liquid chromatography coupled tandem mass spectrometry analysis of endocannabinoids was performed as previously described (Gouveia-Figueira and Nording, 2015), with some modifications. Briefly, mass spectrometric analysis was performed on an Agilent 6490 triple quadrupole mass spectrometer in positive ionization mode. Calibration standards were analyzed over a range of concentrations from 0.025 to 50 pg on column for all of the ethanolamides and 2.5 to 5000 pg on column for the 2-AG. The following lipids were quantified: 2-arachidonyl glycerol (2-AG), arachidonoyl ethanolamide (AEA), docosahexaenoyl ethanolamide (DHEa), docosatetraenoyl ethanolamide (DEA), linoleoyl ethanolamide (LEA), oleoyl ethanolamide (OEA), palmitoleoyl ethanolamide (POEA), palmitoyl ethanolamide (PEA), stearoyl ethanolamide (SEA). Quantitation of endocannabinoids was performed using Agilent Masshunter Quantitative Analysis software. All results were normalized to protein concentration.

### Quantitative real-time PCR

mRNA was purified using the RNeasy Plus Mini kit (QIAGEN) according to the manufacturer’s instructions. RNA was reverse transcribed into cDNA using the High-Capacity cDNA Reverse Transcription kit (Applied Biosystems). Quantitative real-time PCR (RT-qPCR) was performed using the QuantStudio 7 Flex (Applied Biosystems) using TaqMan Fast Advanced master mix and TaqMan primer/probes (Invitrogen) for GAPDH (Rn01775763_g1), FAAH (Rn00577086_m1), CB1 (Rn02758689_s1). Expression data were calculated as fold gene expression using 2^–ΔΔCt^ method with GAPDH as the reference gene, and normalized to untreated control. Four independent technical replicates were performed in each individual experiment.

### Multielectrode array

Dissociated rat cortical neurons were prepared as above and plated at 120,000 cells/well onto 24-well Cytoview plates (Axion Biosystems) coated with 0.1 mg/ml PLL (Sigma-Aldrich). The plating area fully covered the bottom of the well. Recordings were performed. Neurons were maintained as above until DIV21 when they were treated with drugs and used for experiments. Multielectrode array (MEA) recordings were performed in culture media using a Maestro Edge system and AxIS software (Axion Biosystem; version 3.2), with a bandwidth filter from 200 Hz to 3.0 kHz. Spike detection was computed with an adaptive threshold of six times the standard deviation of the estimated noise for each electrode. Plates were left untouched in the Maestro instrument for 10 min before recording, which proceeded for 10 min once every 30 min for as long as needed in each experiment. Data were analyzed using the neural metrics tool (Axion Biosystems; version 3.1.7), under the conditions that an electrode was deemed active if 5 or more spikes occurred over 1 min. The mean firing rate for each well was defined as number of spikes divided by the duration of the analysis, in Hertz. Single electrode bursts were defined as bursts of a minimum of five spikes on one electrode with a maximum interspike interval (ISI) of 100 ms, which was divided by the duration of the analysis to yield burst frequency, in Hertz. Network bursts were defined as bursts of a minimum of 35 spikes that occurred in >35% of the active electrodes in the well, with a maximum ISI of 100 ms, which was divided by the duration of the analysis to yield network burst frequency, in Hertz.

### Transfection and live-cell wide field microscopy

A mixture of 1.5 μl of Lipofectamine 2000 (Invitrogen) and 2.0 μg of pAAV.CAG.SF-iGluSnFR.A184S (Addgene plasmid #106198; gifted from Loren Looger; http://n2t.net/addgene:106198; RRID: Addgene_106198) in 60 μl Neurobasal Medium (Invitrogen) was prepared and incubated at room temperature for 30 min. Media from neuron cultures was saved aside and cells were incubated with this mixture at 37°C and 5% CO_2_ for 30 min. Mixture was aspirated and replaced with original media. Time lapse wide field fluorescence videos were obtained at five frames per second for 60 s using a Zeiss Laser Scanning Microscope (LSM) 800/Airyscan equipped with a Colibri 7 LED light source, Zeiss Axiocam 506 CCD camera, and 63×/1.4 NA objective lens at 37°C and 5% CO_2_. Neurons were imaged in their culture media.

### Live-cell imaging data analysis

Timelapse images were analyzed in FIJI by first subtracting background fluorescence measured from an empty region of the field of view then running an exponential fit bleach correction using a region of interest containing dendrites of a cell in the field of view. The dendritic arbor was traced in FIJI and designated the region of interest. Fluorescence levels in this region of interest were measured and analyzed semi-automatically with a custom code in R, which identifies frames where synchronized fluorescent changes began by locating peaks in the second derivative of a plot of fluorescence over time. A baseline fluorescence was assigned to each synchronous event, corresponding with the fluorescence intensity value of the first frame preceding the synchronous event and change in fluorescence over background (ΔF/F) over baseline was calculated for each frame. The area under the curve of synchronous events was measured in arbitrary units, by summing the ΔF/F values for each frame from the initial rise from baseline to the return to baseline. Interevent interval was calculated by counting the number of frames from the start of one event to the start of the next and converting this value to seconds based on recording frame rate. Statistics were run in Prism. For presentation purposes only, a double derivative time lapse was generated that allows for the clear visualization the change in fluorescence with time.

### Study design and statistical analysis

Results are presented as mean ± SEM from at least three independent culture preparations. Each individual well is treated as an independent biological replicate in statistics (*n* = 1). For the bar charts in Figures 4*D* and 5*C*, all timepoints for one well were binned as one independent replicate in statistics (*n* = 1). Statistical significances were determined using unpaired *t* tests between Ctrl, BCC and TTX. One-way ANOVA and appropriate *post hoc* multiple comparisons were used, as indicated in figure legends, for comparisons of mRNA, protein and targeted lipidomics at different time points in downscaling time course, for comparisons between Ctrl and BCC with or without MTEP/JNJ or AM251, for comparisons of iGluSnFR interevent intervals and area under curve before and after acute AM251 treatment, for comparisons of average network activities for the first 8 h between Ctrl, BCC, and BCC+PF3845-treated cells, and for the comparisons of average network activities for the first 1.5 h between naive and downscaled cells treated with AM251. Two-way ANOVA and appropriate *post hoc* multiple comparisons were used, as indicated in figure legends, for comparisons of network activities between cells treated with Ctrl, BCC and BCC+PF3845, and for comparisons of network activities between naive and downscaled cells treated with AM251. All statistical analyses were performed in GraphPad Prism.

## Results

### Adaptation of the eCB system during homeostatic downscaling

The endocannabinoid system (eCB) is a major neuromodulatory system that regulates neurotransmitter release at both glutamatergic and GABAergic synapses. To determine a possible role of the eCB system in homeostatic plasticity, developing (DIV11–DIV12) and mature (DIV21–DIV25) cultured rat cortical neurons were treated with bicuculline (BCC) or tetrodotoxin (TTX) for 48 h, treatments previously shown to robustly induce homeostatic downscaling and upscaling, respectively (Turrigiano et al., 1998). Adjustments in surface AMPAR receptor density and the phosphorylation status of AMPAR subunit GluA1 at S845 are previously described markers for synaptic scaling (O’Brien et al., 1998; Hu et al., 2010; Diering et al., 2014). Neurons were surface biotinylated followed by lysis to allow for the detection of total and surface proteins (Fig. 1*A*). We examined the expression of AMPAR receptor subunit GluA1 and cannabinoid receptor 1 (CB1). In developing and mature cultures, BCC induced a significant downregulation in surface GluA1 and dephosphorylation of GluA1 S845 (pS845), indicating that the neurons have engaged homeostatic downscaling, consistent with previous findings (Fig. 1*A–C*). Network suppression with TTX caused a trend of increased surface GluA1 as expected, although these changes were not significant, and in mature cultures we observed the expected TTX-induced increase in phosphorylation of GluA1 S845 previously associated with homeostatic upscaling (Diering et al., 2014; Kim and Ziff, 2014). In developing cultures BCC had no effect on CB1 expression, whereas TTX treatment caused a significant increase in surface, but not total, CB1 levels (Fig. 1*B*). In contrast, mature cultures responded to BCC by a significant upregulation of surface and total CB1 expression, compared with control, whereas TTX had no effect (Fig. 1*C*). The above data suggest that the eCB system adapts to changes in network activity in a manner that depends on the maturation of the culture.

To further investigate the maturational changes *in vitro*, we measured synapse and eCB system protein expression in neurons at DIV8, DIV15, and DIV22 using Western blotting (Fig. 1*D*). In the second week *in vitro* (DIV8 vs DIV15), we found a dramatic increase in all proteins examined (Fig. 1*E*). In the third week *in vitro* (DIV22 vs DIV15), we found a striking increase in the expression of vesicular glutamate transporter 1 (vGluT1) and fatty acid amid hydrolase (FAAH), the principal degradative lipase for anandamide (AEA) and related N-acylethanolamines (NAEs). Additionally, we found a moderate increase in GluA1, PSD95, and vesicular GABA transporter (vGAT) and no change in CB1 (Fig. 1*E*). These results suggest that, compared with developing neurons, mature neurons further undergo a significant amount of synapse maturation, especially in presynaptic glutamate release machinery such as vGluT1, and potentially have significantly different metabolism of NAEs as indicated by increased expression of FAAH. In our subsequent experiments we have focused on the adaptation of the eCB system to network hyperexcitability in mature neuron cultures.

### Temporal dynamics of eCB adaptations

To further characterize the adaptations in the eCB system during homeostatic downscaling in mature cultures, we performed a time course of BCC treatment (4–48 h) followed by RT-qPCR and Western blot analysis of transcript and protein expression, respectively, and quantification of eCB lipids using targeted mass spectrometry. Total GluA1 showed a significant decrease beginning at 24 h as expected. pGluA1-S845 increased over the first 12 h but decreased significantly at 48 h (Fig. 2*A*,*B*). CB1 protein increased significantly but only at 48 h, suggesting that this was a delayed response (Fig. 2*B*). Interestingly, FAAH protein decreased progressively starting at 12 h and remained significantly downregulated through the 48-h time course, suggesting that adaptations in eCB metabolism are an earlier step in homeostatic downscaling (Fig. 2*B*). Accordingly, targeted mass spectrometry of eCBs showed that while 2-AG upregulation was a prominent feature of BCC treatment at 4 h, a significant upregulation of AEA became the dominant response at 24 h and onward. By 48 h, multiple NAEs, all of which are FAAH substrates, were significantly upregulated (Fig. 2*C*). The BCC-induced decrease in FAAH protein was matched by a decrease in the expression of FAAH mRNA at 12–24 h, suggesting that changes in FAAH protein are controlled in part at the level of transcription. Interestingly, BCC treatment induced an upregulation of CB1 mRNA at 12–24 h post-BCC treatment, in advance of CB1 protein increases at 48 h (Fig. 4*D*). Together, these findings suggest that during the induction and expression of homeostatic downscaling in mature cortical cultures, there is a stepwise adaptation in the eCB system starting with downregulation of FAAH protein and consequent increases in the abundance of AEA and other NAEs, followed by the delayed upregulation of CB1. Changes in AEA metabolism and upregulation of CB1 likely synergize to enhance tonic eCB signaling.

### Coordination of presynaptic CB1 signaling and postsynaptic glutamatergic signaling

Homeostatic downscaling in cultured rodent neurons is primarily understood as a postsynaptic adaptation that involves downregulation of synaptic AMPARs. Since BCC-induced upregulation of CB1 receptors suggests a coordinated presynaptic and postsynaptic response, we next performed a series of pharmacology experiments to examine the relationship between postsynaptic glutamatergic signaling and presynaptic cannabinergic signaling during BCC-induced downscaling. A previous report has shown that hyperexcitation caused by BCC resulted in activation of type I mGluR1/5 and that a cocktail of noncompetitive mGluR1/5 inhibitors could prevent postsynaptic downscaling of AMPARs (Hu et al., 2010). We confirmed this finding by treating neurons with a cocktail of 1 μm MTEP and 100 nm JNJ16259685 (MTEP/JNJ), noncompetitive inhibitors of mGluR5 and mGluR1, respectively, and showing that this treatment prevented BCC-induced downregulation of surface GluA1 and partially blocked the dephosphorylation of pS845 (Fig. 3*B*). However, MTEP/JNJ treatment had no effect on BCC-induced upregulation of total and surface CB1 (Fig. 3*B*). This result suggests that presynaptic adaptation of CB1 during downscaling is independent of postsynaptic mGluR1/5-mediated signaling.

Next, we blocked cannabinergic signaling in cultured rat cortical neurons by treating with selective CB1 antagonist AM251 (500 nm) and examined the effect on postsynaptic adaptation during downscaling. AM251 treatment had no effect on surface GluA1 and pS845 and did not prevent the BCC-induced downregulation of surface GluA1 and pS845 (Fig. 3*D*). Interestingly, the BCC-induced upregulation of CB1 was not prevented by AM251, suggesting that CB1 upregulation is also independent of CB1 signaling. Together, these findings suggest that neurons respond to network hyperexcitability with coordinated cannabinergic and glutamatergic adaptations, which occur through independent molecular mechanisms.

### Enhancing eCB signaling suppresses network activity during hyperexcitation

In the context of network activity, CB1 signaling is well characterized to regulate presynaptic vesicle release dynamics (Castillo et al., 2012), and to maintain excitatory up-states, an intrinsic mode of cortical network activity particularly prominent in sleeping and anesthetized animals (Steriade et al., 1993a; Pava et al., 2014). Since up-state like network activity is also well documented in organotypic slices (Sanchez-Vives and McCormick, 2000; Johnson and Buonomano, 2007) and cultured cortical neurons (Kamioka et al., 1996; Canepari et al., 1997; Saberi-Moghadam et al., 2018), the reorganization in eCB system described above (Fig. 2*A–D*) may be involved in the regulation of network activity *in vitro*. To better understand the role of eCBs in network activity *in vitro*, we monitored spiking activities from neurons plated on multielectrode arrays (MEAs) as neural networks adapt to BCC-induced hyperexcitation (Fig. 4*A*). Mean firing rate was quantified as total number of spikes divided by the duration of the analysis. Single electrode bursts (Fig. 4*B*, red line) and networks bursts (Fig. 4*B*, dotted black box) were identified based on spike densities and participating electrodes (see Materials and Methods), the total of which was then divided by the duration of the analysis to yield burst frequency and network burst frequency, respectively.

Baseline *in vitro* network activity was first measured in cultured neurons grown on MEA plates, and then changes in activity were monitored longitudinally following the addition of BCC or pharmacological manipulations of the eCB system. In agreement with previous studies, we observed that mature neurons (>DIV21) exhibited baseline levels of spontaneous network activation characterized by highly rhythmic windows of coordinated firing (“network bursts”) followed by periods of relative network silence (Fig. 4*B*, control condition). BCC application rapidly (<10 min) converted the *in vitro* network activity to a burst firing pattern (Fig. 4*B*,*C*). Following BCC treatment, the mean firing rate and network burst frequency peaked around 1–2 h and remained significantly elevated for ∼8 h, after which these measures of network activity became statistically indistinguishable from the baseline, suggesting that some homeostatic adaptations had occurred following 8 h of BCC treatment (Fig. 4*C*, top, bottom, dotted gray vs solid gray, #*p* < 0.05). Similarly, the single-electrode burst frequency also peaked around 1–2 h but remained significantly elevated for 20 h (Fig. 4*C*, middle, dotted gray vs solid gray, #*p* < 0.05). After chronic BCC treatment (>24 h), *in vitro* network activity recovers to a level statistically indistinguishable from baseline but is not lowered further (Fig. 4*C*, dotted gray vs solid gray). The recovery of *in vitro* network activity within 8 h of hyperexcitation onset likely reflects mechanisms of homeostatic processes other than downregulation of synaptic AMPARs and excitatory drive which is usually expressed after chronic (>24 h) perturbations in network activity (Fig. 2*B*; Diering et al., 2014; Fong et al., 2015; Gonzalez-Islas et al., 2020).

Decreases in FAAH could be detected as early as 12 h (Fig. 2*B*), therefore, we hypothesized that the downregulation of FAAH and upregulation of AEA may be involved in network adaptation to BCC-induced hyperactivity. Accordingly, increasing AEA/NAE availability by application of selective FAAH inhibitor PF3845 (Ahn et al., 2009) suppressed BCC-induced network hyperexcitation in a dose-dependent manner, resulting in lowered mean firing rate, burst frequency and network burst frequency comparing to BCC only treatment (Fig. 4*C*, *p < 0.05, $*p* < 0.05). Notably, FAAH blockade strongly suppressed network activity on immediate application (<10 min), even at subsaturating dosage (100 nm), suggesting that AEA metabolic turnover is rapid and under tight regulation. The dose-dependent suppression of hyperexcitation is particularly prominent during the first 8 h of BCC treatment (Fig. 4*D*). This result suggests that downregulation of FAAH, and upregulation of AEA, is part of a mechanism to reduce network excitability in response to BCC-induced hyperexcitation.

### Inhibiting CB1 signaling and excitatory network activity

Since the eCB system is involved in the regulation of *in vitro* network activity (Fig. 4*C*,*D*), we hypothesized that the delayed upregulation of CB1 after BCC treatment described above (Figs. 1*C*, 2*B*) predicts a different network response to CB1 antagonism before and after 48 h BCC treatment. To examine the role of CB1 on *in vitro* network activity following chronic BCC treatment, we left neurons untreated (naive) or treated with BCC (downscaled) for 40 h, and then monitored changes in spiking activities during 6 h of CB1 inhibition using 50 nm AM251 (Fig. 5*A*). In naive cells, CB1 antagonism consistently led to a transient but significant decrease in all activity metrics examined for the first 1.5 h, indicating acute suppression of *in vitro* network activity (Fig. 5*B*, dotted gray vs dotted black #*p* < 0.05, and Fig. 5*C*). In contrast, AM251 treatment in downscaled cells resulted in minimal effects on measures of *in vitro* network activity (Fig. 5*B*, solid gray vs solid black, and Fig. 5*C*). The weakened effect of AM251 on mean firing rate and burst frequency in downscaled cells is sustained for at least 6 h (Fig. 5*B*, dotted black vs solid black, *p < 0.05).

Since CB1 is known to regulate the release of neurotransmitters including glutamate, we examined whether changes in glutamate release were involved in network adaptations. To this end, we indirectly measured excitatory network activities *in vitro* by visualizing glutamate release dynamics with a high-affinity variant of fluorescent glutamate biosensor iGluSnFR-A184S (Marvin et al., 2013, 2018). iGluSnFR is a plasma-membrane-targeted fluorescent reporter that responds to extracellular glutamate release with an increase in fluorescence (Marvin et al., 2018). We expressed iGluSnFR in mature neurons (>DIV21) using lipofection and examined glutamate release dynamics in live-cells using wide-field microscopy time-lapse recording. Glutamate release is quantified as a transient increase in iGluSnFR fluorescence (ΔF/F).

Under control conditions, we observed spontaneous synchronous events where fluorescence across the entire dendritic arbor was increased (Fig. 5*D*). The “wave-like” synchronous events were highly rhythmic, reminiscent of *in vitro* network bursts recorded by MEA. Indeed, synchronous iGluSnFR events were completely suppressed by the sodium channel blocker TTX and reduced in amplitude by the NMDAR inhibitor D-AP5 (Extended Data Fig. 5-1) matching previously reported effects of these drugs on *in vitro* network events (Steriade et al., 1993b; Sanchez-Vives and McCormick, 2000). Therefore, we suggest that synchronous iGluSnFR events are analogous to “cortical up-sate like” events measured by MEA as *in vitro* network bursts. Moreover, acute BCC treatment (∼10 min) resulted in a plateau in iGluSnFR synchronous events, suggesting a transiently sustained glutamate release indicative of hyperexcitation (Extended Data Fig. 5-1), consistent with the transient increase in bursting activity measured by MEA recordings (Fig. 4*B*,*C*). Following 48 h chronic BCC treatment, the plateau of synchronous events was absent, and the frequency and amplitude of synchronous events were comparable to control level, indicative of homeostatic adjustment of synchronous glutamate release (Fig. 5*E*,*F*). The similar pharmacological responses and homeostatic adjustment likely reflect that the synchronous glutamatergic events measured by iGluSnFR imaging are, to a certain degree, analogous to network events detected by MEA.

To examine the effect of CB1 antagonism on glutamate release following the induction of downscaling, we acutely blocked CB1 with AM251 for 1 h in naive, or BCC-treated neurons. In both naive and downscaled cells, acute AM251 significantly suppressed the magnitude of synchronous glutamate release events (Fig. 5*E*), as measured by decreased area under the curve (Fig. 5*F*, right). Further, in naive cells CB1 inhibition reduced the frequency of glutamatergic events, whereas in downscaled cells, AM251 application caused glutamatergic events of low amplitude to become more frequent (Fig. 5*E*,*F*, left). Together, these data suggest that CB1 inhibition has a diminished capacity to suppress *in vitro* network events after downscaling.

## Discussion

**Figure 6.**
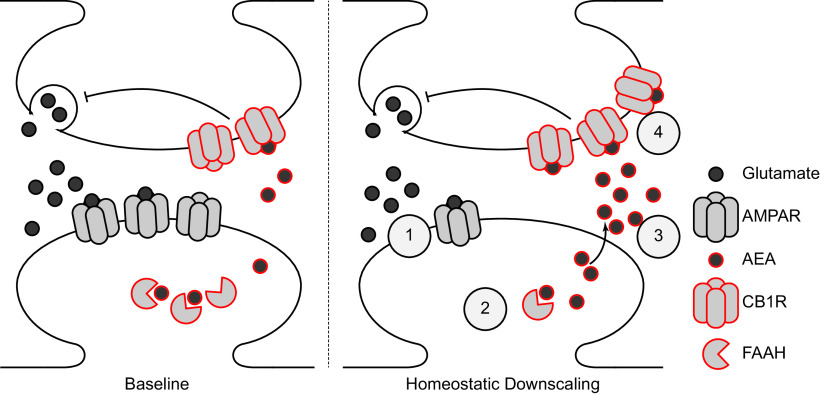
Model of homeostatic adaptation of the endocannabinoid system during downscaling. BCC-induced downscaling drives coordinated remodeling of synapses: (1) downregulation of surface AMPARs, (2) downregulation of FAAH, (3) accumulation of FAAH substrates including AEA, and (4) upregulation of surface CB1 receptors. Elevated FAAH substrates may serve to suppress network during adaptation to hyperexcitation, but CB1 signaling is also required to maintain network activities at baseline.

The eCB system has been well described to participate in multiple forms of short-term and long-term synaptic plasticity (Castillo et al., 2012). However, to our knowledge, homeostatic adaptations of the eCB system *in vitro* have only been studied in the context of chronic network silence (Kim and Alger, 2010; Song et al., 2015). In this study we provide quantitative analysis of the adaptations of the eCB system to network hyperexcitability induced by chronic BCC treatment in mature cultured rat cortical neurons, an *in vitro* system that has been used to investigate the molecular mechanisms of homeostatic downscaling (Turrigiano et al., 1998; Hu et al., 2010; Diering et al., 2014). We report evidence of rapid and persistent downregulation of FAAH, consequent upregulation of bioactive NAEs including AEA, and delayed upregulation of CB1. Furthermore, we show that presynaptic cannabinergic adaptations and postsynaptic glutamatergic adaptations occur through independent mechanisms. Finally, we show that endocannabinoid signaling through CB1 stabilizes *in vitro* network activities during homeostatic adaptation to hyperexcitation (Fig. 6).

### Mechanisms of homeostatic adaptation to hyperexcitation in cultured neurons

Homeostatic downscaling was induced successfully by prolonged (48 h) GABA antagonism (BCC) in developing (∼DIV14) and mature (>DIV21) neuron cultures, as evidenced by downregulation of surface GluA1 and dephosphorylation of pGluA1-S845 (Fig. 1*B*,*C*), matching previous reports (O’Brien et al., 1998; Diering et al., 2014). Distinct molecular mechanisms have been shown to underlie the initial “induction” and sustained “expression” of homeostatic downscaling. Induction involves Erk1/2-dependent activation of scaling factors Arc and Homer1a and subsequent activation of ligand-independent signaling from mGluR1/5 within 1–12 h of BCC treatment (Hu et al., 2010; Bateup et al., 2013). Expression involves downregulation of total and surface AMPARs and dephosphorylation of pGluA1-S845 (O’Brien et al., 1998; Diering et al., 2014). Notably, BCC treatment appears to regulate pGluA1-S845 in a biphasic manner (Fig. 2*B*), similar to the biphasic network responses observed on MEA recordings (Fig. 4*C*) consistent with GluA1-S845 dephosphorylation being a component of scaling expression rather than induction. Here, we show that BCC treatment drives adaptations in the eCB system by regulating FAAH and CB1 mRNA levels by 12 h followed by a delayed upregulation of CB1 total and surface protein by 48 h. This time course suggests that coordinated adaptations to the eCB system may similarly constitute mechanisms of downscaling expression, rather than induction. Additional research will be needed to fully understand the role of eCB adaptations in homeostatic plasticity. Homeostatic upscaling is typically induced by chronic (48 h) network silencing in response to TTX (Turrigiano, 2008) which we show resulted in the expected hyperphosphorylation of GluA1-S845 (Diering et al., 2014; Kim and Ziff, 2014), but had no effect on CB1 surface or total protein levels in mature cultures. However, in our hands, TTX did not robustly drive the expected increase in surface GluA1 levels. Therefore, we refrain from making any conclusions on the adaptations to the eCB system in homeostatic upscaling, and instead focus only on downscaling given its implication in the neurocognitive benefits of restorative sleep (Tononi and Cirelli, 2003, 2014; Diering et al., 2017; Puentes-Mestril and Aton, 2017). Additional optimizations, such as upscaling induction with co-treatment of TTX and NMDA antagonists (Bülow et al., 2019), may enable further investigations into the role of eCB system in upscaling.

Since BCC is well characterized to induce extensive remodeling of synaptic proteome and phosphoproteome to drive compensatory adjustments in postsynaptic AMPAR density (Wu et al., 2022), it is reasonable to postulate that overlapping mechanisms and similar receptor post-translational modifications drive endocannabinoid adaptations. Here, we show that one such candidate mechanism, mGluR1/5, a prominent signaling hub of activity-dependent plasticity, is independent of endocannabinoid adaptation (Fig. 3). Other candidate mechanisms may be speculated based on recent studies. CB1 contains multiple serine residues that are putative substrates of phosphorylation by protein kinase C (PKC; Eldeeb et al., 2019), a phosphorylation pathway implicated in homeostatic downscaling in cultured rat hippocampal neurons (Schanzenbächer et al., 2016; Dörrbaum et al., 2020). Moreover, CB1 contains an interaction motif in C-terminal helix 9 that has been shown to mediate CB1 protein interactions important for CB1 axonal targeting and surface expression (Fletcher-Jones et al., 2019, 2020). Further studies examining the activity-dependent post-translational modification of CB1 and regulation of CB1 interacting proteins are needed to understand the mechanisms by which downscaling stabilizes CB1 surface expression.

### *In vitro* developmental regulation of the homeostatic response

In developing cultures approximately two weeks *in vitro*, homeostatic plasticity predominantly reflects changes in postsynaptic receptor density (Turrigiano, 2008; Diering et al., 2014). Additional presynaptic homeostatic adaptations are engaged as neurons mature in culture approximately three weeks *in vitro* (Wierenga et al., 2006). Similarly, our data show that the more mature three-week-old neuron cultures respond to chronic hyperexcitation in part through upregulation of surface and total CB1 (Fig. 1). Other adaptations in mature cultures include homeostatic regulation of vesicular transporters (De Gois et al., 2005). Compared with developing cultures, mature cultures undergo further synaptic, dendritic, and functional development, which may confer greater need to regulate presynaptic components and network activities during homeostatic downscaling (Wagenaar et al., 2006; Harrill et al., 2015). Mature cultures show more developed synapses as evidenced by increased expression of postsynaptic ion channel GluA1 and presynaptic vesicle release machineries vGluT1 and vGAT (Fig. 1*E*). Although total CB1 levels were similar at DIV15 and DIV21, we observed a clear increase in the expression of FAAH, indicating that the eCB system is still undergoing maturation. We suggest that the upregulation of FAAH, and the emergence of the BCC-induced upregulation of CB1 during the maturation of cortical neurons *in vitro*, may reflect the developmental emergence of the need for activity-dependent regulation of synchronous network activities (Kamioka et al., 1996; Johnson and Buonomano, 2007).

### 2-AG versus AEA

2-AG and AEA are both well-described agonists of the CB1 receptor. However, these two metabolites have completely nonoverlapping enzymes for synthesis and degradation, suggesting that the two most prominent eCBs undergo distinct regulation and serve separate physiological functions (Di Marzo, 2018). 2-AG mediates major forms of eCB-dependent plasticity, such as DSI, DSE and mGluR-dependent LTD (Tanimura et al., 2010; Yoshino et al., 2011; Castillo et al., 2012). However, AEA has been implicated in cortical up-sate activity, sleep stability and memory (Murillo-Rodríguez et al., 1998; Busquets-Garcia et al., 2011; Pava et al., 2014), and AEA but not 2-AG are increased in cortex during the sleep phase (Martin et al., 2022). Our results indicate that AEA but not 2-AG exerts tonic action during homeostatic downscaling (Fig. 2). Some properties make AEA more suitable for tonic signaling. First, AEA is considered a partial agonist of CB1, which makes it less efficacious than full agonists such as 2-AG (Sugiura et al., 1996; Burkey et al., 1997; Glass and Northup, 1999). By regulating AEA during global downscaling of synaptic strength, neurons may preserve sensitivity to 2-AG-mediated “fine-tuning” of individual synapses. Second, sustained 2-AG signaling results in desensitization of CB1, whereas sustained AEA signaling does not (Schlosburg et al., 2010). By minimizing receptor desensitization, neurons may maintain tonic endocannabinoid signaling throughout the relatively long time frame of homeostatic plasticity and avoid functional antagonism because of sustained 2-AG signaling, while at the same time preserving the short-term plasticity mediated by 2-AG. Indeed, Kim and Alger (2010) showed that chronic inactivity in hippocampal slices reduces endocannabinoid tone by regulating AEA degradation instead of 2-AG. Therefore, we hypothesize that 2-AG and AEA indeed mediate distinct mechanisms: a phasic action of 2-AG mediating short-term plasticity, and a tonic action of AEA that regulates neuronal network activity over longer time scales.

Our time course data suggest that multiple FAAH substrates have become significantly upregulated by 48 h (Fig. 2*C*). Since FAAH is the major enzyme for catabolizing multiple endocannabinoid substrates in the NAE family, it is possible that cellular levels of NAEs are regulated through a common mechanism to mediate coordinated signaling events (Di Marzo, 2018). Two such NAEs, oleoylethanolamide (OEA) and palmitoylethanolamide (PEA), are agonists for nuclear endocannabinoid receptor PPARα, and have been implicated in sleep-wake regulation and memory acquisition (Mazzola et al., 2009; Murillo-Rodríguez et al., 2011). Indeed, PPARα has been described to be required for expression of the immediate early gene Arc in response to hippocampal neuronal activity through CREB-mediated transcriptional control of plasticity-related molecules (Roy et al., 2013). Thus, we speculate that AEA-CB1 signaling is only one part of a coordinated neuronal response that synergizes with signaling pathways activated by other bioactive NAEs. A possible role for OEA, PEA, and/or PPARα should be investigated in future studies.

### CB1, cortical up-states, and NREM sleep

In the absence of external stimuli, such as during anesthetization and NREM sleep, the mammalian neocortex shows intrinsic network oscillation between periods of synchronized depolarization (up-state) and prolonged network silence (down-state; Steriade et al., 1993b; Timofeev et al., 2001). Cortical slice preparations and dissociated cultures are also known to exhibit NREM like up-state synchronous activity (Kaufman et al., 2014; Pava et al., 2014; Saberi-Moghadam et al., 2018), replicated here as network bursts by MEA recording (Fig. 4) or synchronous glutamatergic activity measured by iGluSnFR live-cell imaging (Fig. 5). Emerging research shows that increased eCB signaling can promote sleep behavior by stabilizing NREM episodes and supporting cortical up-states (Pava et al., 2014; Pava et al., 2016; Kesner and Lovinger, 2020), while inhibition of CB1 causes sleep fragmentation (Martin et al., 2022). Further, we recently showed that AEA, and not 2-AG, is regulated during the wake sleep cycle in cortical synapse fractions (Martin et al., 2022). Here, we show that coordinated remodeling of AEA-CB1 signaling is important to stabilize *in vitro* oscillatory network activity during homeostatic downscaling. Finally, remodeling of cortical synapses through homeostatic downscaling has been proposed to mediate part of the neurocognitive benefits of sleep (Tononi and Cirelli, 2014; de Vivo et al., 2017; Diering et al., 2017). Therefore, we speculate that the adaptations to the AEA-CB1 system during homeostatic downscaling described here *in vitro*, are relevant to furthering our understanding of the molecular basis for the restorative benefits of sleep. Considerably more research will be needed to fully describe the molecular mechanisms underlying the adaptations of the eCB system during homeostatic plasticity and the links to *in vivo* physiological functions.

## Synthesis

Reviewing Editor: Eran Perlson, Tel Aviv University Decisions are customarily a result of the Reviewing Editor and the peer reviewers coming together and discussing their recommendations until a consensus is reached. When revisions are invited, a fact-based synthesis statement explaining their decision and outlining what is needed to prepare a revision will be listed below. The following reviewer(s) agreed to reveal their identity: Seonil Kim, Stephen Smith.

The authors present a compelling set of experiments showing that the endocannabinoid system is involved in a well-established in vitro model of homeostatic plasticity, using rat cortical neuron cultures. They demonstrate that the enzyme responsible for degradation of anandamide, and the CB1 receptor, are up/downregulated, respectively, following 48hrs of BIC treatment; they show that CB1 upregulation is independent of mGluR or CB signaling; and they show that increasing or decreasing CB tone using inhibitors of FAAH or antagonists of CB1 has different effects on the electrophysiology and glutamate release of BIC-exposed neurons compared to naïve controls.

Specific comments to be addressed:

1. The authors should include the discussion of the potential mechanisms that link between a BCC-induced decrease in neuronal activity and the endocannabinoids system. How does BCC regulate CB1 receptors and FAAH activity?

2. The manuscript would be improved to include the discussion of why TTX treatment in mature neurons was unable to induce upscaling in Fig. 1.

3. In Fig. 2B, BCC treatment appears to regulate pGluA1-S845 levels over time in a bi-phasic manner. The authors need to include the explanation.

4. Would chronic BCC treatment (>24 hrs) decrease in vitro cortical network activity in Fig. 4?

5. The authors should use “in vitro” oscillatory activity to distinguish “in vivo” network activity.

6. The authors use several different “hours” expressions throughout the text (e.g. hr, hours, h, and so on).

7)For all figures, I would prefer to see bar graphs with individual values included. This would also clarify weather triplicate wells were treated as a single N or 3 three independent replicates in the statistics.

8)The blot of CB1 in figure 1D looks overexposed beyond any linear range of the detector for DIV15 vs DIV22- all 4 bands are saturated. Are there less-exposed blots?

9)"suggesting that CB1 protein upregulation is controlled by both transcriptional and translational regulation"- this is not supported by the data. A lag in protein upregulation following mRNA upregulation is to be expected if the mRNA copy number is low, or the rate of protein accumulation is slow, etc, since the detection of mRNA vs protein is much more sensitive.

10)I would prefer if the authors referred to the mechanism of CB1 regulation as “mGluR-independent", rather than “independent of postsynaptic glutamatergic scaling”. Some components of AMPA vs CB regulation may be identical- like perhaps CAMKII dependence?

11)Figure 3C-D: What about FAAH?

12)Page 18: “decreases in AEA could be detected early on (Fig 2B)”: decreases in FAAH were detected at 12 hours, is that what the authors are referring to?

13)Fig 4: it is striking how FAAH blockade severely reduced network activity at 10min, even in the very low dose. The authors should comment on this.

14)I think the authors should tone down the analogies to “cortical upstates” (e.g. Page 20, but also elsewhere). They are measuring in vitro plasticity and glutamate release; they do not need to extrapolate to anything more than that.
